# Risk factors of laryngeal injuries in extubated critical pediatric patients

**DOI:** 10.1186/s43054-021-00064-0

**Published:** 2021-07-28

**Authors:** HebatAllah Fadel Algebaly, Mona Mohsen, Maggie Louis Naguib, Hafez Bazaraa, Noran Hazem, Miriam Magdy Aziz

**Affiliations:** Department of Pediatrics, Faculty of Medicine, Cairo University, Cairo, Egypt

**Keywords:** Laryngeal injury, Post-extubation, Flexible laryngobronchoscopy, Pediatrics

## Abstract

**Background:**

The larynx in children is unique compared to adults. This makes the larynx more prone to trauma during intubation. Under sedation and frequent repositioning of the tube are recorded as risk factors for laryngeal injury. We examined the larynx of 40 critically ill children in the first 24 h after extubation to estimate the frequency and analyze the risk factors for laryngeal trauma using the classification system for acute laryngeal injury (CALI).

**Results:**

The post-extubation stridor patients had a higher frequency of diagnosis of inborn errors of metabolism, longer duration of ventilation, longer hospital stay, moderate to severe involvement of glottic and subglottic area, frequent intubation attempts, and more than 60 s to intubate Regression analysis of the risk factors of severity of the injury has shown that development of ventilator-associated pneumonia carried the highest risk (OR 32.111 95% CI 5.660 to 182.176), followed by time elapsed till intubation in seconds (OR 11.836, 95% CI 2.889 to 48.490), number of intubation attempts (OR 10.8, CI 2.433 to 47.847), and development of pneumothorax (OR 10.231, 95% CI 1.12 to 93.3).

**Conclusion:**

The incidence of intubation-related laryngeal trauma in pediatric ICU is high and varies widely from mild, non-symptomatic to moderate, and severe and could be predicted by any of the following: prolonged days of ventilation, pneumothorax, multiple tube changes, or difficult intubation.

## Background

Tracheal intubation (TI) is a life-saving procedure in critically ill pediatrics [1]. Critically ill children have difficult airway anatomy, low oxygen reserve that places them at high risk for tracheal intubation-associated complications [2]. The occurrence of TI-associated events was significantly associated with longer mechanical ventilation duration (MV) [3]. The endotracheal tube exerts pressure on the posterior aspect of the laryngeal mucosa; the resulting ischemia causes post-intubation acute laryngeal lesions [4]. Risk factors for the development of laryngeal trauma include preexisting tracheal irritation, upper airway infection, incorrect size of the endotracheal tube, too high cuff pressure, traumatic intubation, repeated intubation attempts, prolonged intubation period, too aggressive tracheal aspiration, tube mobility, and patient fighting against endotracheal intubation [5]. A metaanalysis in 2018 recorded the absence of guidelines establishing for post-extubation assessment that increase the risk of medical sequelae [6].

Flexible fiberoptic laryngobronchoscopy has a sensitivity of 93.7% and a negative predictive value of 98.8% when performed to screen post-extubation laryngeal lesions [7]. The frequency of moderate to severe injuries was reported to range from 33.5% up to 43% in several studies [8–10]. Classifications were adapted by Lindholm [8], Colice [9], and Benjamin [10]. The classification of acute laryngeal injuries CALI classification system was introduced in 2016 and divided the lesions to mild, moderate, and severe and is considered more specific to predict the progression into subglottic stenosis [11]. The assessment of the development of acute airway lesions themselves after intubation could contribute to making way for early interventions and preventive strategies to reduce the long-term risk of subglottic stenosis [12]. In this study, we aimed to evaluate the types of laryngeal lesions and their risk factors in extubated pediatric patients and analyze the relation to extubation success and ventilation duration.

## Methods

This prospective study was conducted in 23-bed medical ICU over a 9-month period. We included patients from 1 month to 8 years who received invasive ventilation for more than 48 h. Self-extubated children and those with known congenital anomalies of the larynx or head and neck surgery were excluded.

All children passed through a successful spontaneous breathing trial with pressure support ventilation of 5 mmHg before planned extubation. All patients were intubated for emergency medical conditions. The intubations were performed by the intensivist. The tube size was chosen based on the formula described by the American Heart Association [13], and infants received adequate sedation with midazolam (50 mic/kg/dose) and fentanyl (1 mcg/kg/dose) during mechanical ventilation.

Laryngeal nasofibroscopy examination was performed by the pediatric pulmonologist within 24 h after extubation. We examined the larynx of 40 critical pediatric patients using 3.1-mm flexible fiber optic laryngobronchoscopy manufactured by a Penttax company at the bedside passing through the anterior nares. During the procedure, cardiac monitoring and pulse oximetry were maintained. The gel was applied over the anterior nares and also around the terminal end of the scope for smooth and non-traumatic passage through the nose. No sedation was used and oxygen was supplied via mask while maintaining airway position and suction. For most patients, local lidocaine was applied via the bronchoscope channel when approaching vocal cords. The fiberoptic laryngoscope was introduced to the supraglottic region, and images of the supraglottic, glottic, and subglottic regions were obtained, and the findings were stratified according to Schweiger et al. who introduced the CALI classification system for acute laryngeal injuries in pediatric patients after intubation [11].

Data collected as follows: the tube size; the route of intubation; time elapsed till the tube is placed; number of attempts; duration of intubation, whether the tube is placed at day or night; and the complications of invasive ventilation.

Patients were monitored in the 48 h following extubation. Post-extubation stridor (PES) occurrence was noted and whether patients needed reintubation (after programmed extubation).

## Results

### Patients

We performed an upper airway laryngobronchoscopy for 40 extubated pediatric patients in the ICU. The median age was 6 months and the median duration of ventilation was 7 days and IQR (5-11). The most common reason for intubation was pneumonia, then multiple organ system failure (MOSF), followed by coma. Demographic data of the study population are shown in Table 1.

**Table 1:** Demographic characteristics of the study population

Characteristics of the study population	
**Characteristic**	**Median (IQR)**
Female gender	19 (47.5)
Age months	6 (5–11)
Length of stay in ICU in days	16 (8–30)
Duration of ventilation before extubation trial in days	7 (5–10)
**Reason for intubation**	**Number and %**
Pneumonia	23/40 ( 57.5)
Septic shock (MOSF)	9/40 (22.5)
Coma	6/40 (15)
Heart failure	2/40 (5)
**Number of reintubation**	**Number and %**
1	10/40(25)
2	4/40(10)
>2	7/40 (17.5)
**Complications from MV**	**Number and %**
Pneumothorax	8/40 (20%)
Ventilator-associated pneumonia	20/40 (50%)
**Severity by CALI**	**Number and %**
Mild or no injury	20/40 (50)
Moderate to severe injury	20/40 (50)

*ICU* intensive care unit, *MOSF* multiple organ system failure, *MV* mechanical ventilation, *CALI* classification of acute laryngeal injuries

### Post-extubation stridor (PES)

Seventeen patients developed PES. They had a higher frequency of metabolic crises diagnosis, longer duration of ventilation, longer hospital stay, they all have lesions in the glottic and subglottic area by endoscopic examination, frequent intubation attempts, more than a 60-s time needed to intubate, and tendency towards night-time intubation. Characteristics of the patients who developed PES are shown in Table 2 divided into two groups (a group with no or mild lesions and the other with moderate to severe lesions).

**Table 2:** Comparison between two groups as regards clinical characteristic

		Normal or mild lesions by endoscopy	Moderate to severe lesions	***p*** value
**Age median (IQR)**		**6 (4 - 14.5)**	**6.5 (5.5–9.5)**	**0.775**
**Gender: females** **Number (%): males**		**7 (35%)**	**12 (60%)**	**0.113**
		**13 (65%)**	**8 (40%)**	
**Acute pneumonia**		**10 (50.0%)**	**2 (10.0%)**	**0.006**
**CNS disorder**		**2 (10.0%)**	**3 (15.0%)**	**0.632**
**Septic shock**		**1 (5.0%)**	**4 (20.0%)**	**0.151**
**Cardiogenic shock**		**1 (5.0%)**	**2 (10.0%)**	**0.548**
**Emergency surgery**		**3 (15.0%)**	**2 (10.0%)**	**0.632**
**Inborn error of metabolism**		**0 (0.0%)**	**4 (20.0%)**	**0.035**
**Immunodeficiency**		**1 (5.0%)**	**2 (10.0%)**	**0.548**
**Acute compromise in renal patients**		**2 (10.0%)**	**1 (5.0%)**	**0.548**
Days of MV* before 1st trial of extubation, median (IQR)		**5 (4–6)**	**10 (8–12)**	**0.000**
Total duration of MV*, median (IQR)		**5 (2–6)**	**22(6–54)**	**0.057**
**Length of stay in ICU in days, median (IQR)**		**10 (7–12)**	**30 (18–64)**	**0.000**
**Tube size**	**Mean±SD**	**3.95 ± 0.51**	**4.25 ± 0.72**	**0.135**
**Type**	**Oral**	**19 (95.0%)**	**18 (90.0%)**	**0.548**
	**Nasal**	**1 (5.0%)**	**2 (10.0%)**	
**Intubation attempts**	**Once**	**11 (55.0%)**	**2 (10.0%)**	**0.000**
	**Twice**	**9 (45.0%)**	**9 (45.0%)**	
	**Multiple**	**0 (0.0%)**	**9 (45.0%)**	
**Time elapsed**	**< 30 s**	**9 (45.0%)**	**0 (0.0%)**	**0.000**
	**30–60 s**	**8 (40.0%)**	**5 (25.0%)**	
	**> 60 s**	**3 (15.0%)**	**15 (75.0%)**	
**Time of day**	**Day**	**13 (65.0%)**	**7 (35.0%)**	**0.058**
	**Night**	**7 (35.0%)**	**13 (65.0%)**	

### Endoscopic findings

Hyperemia (with or without edema) involving the supraglottic region was present in 33 patients (82.5%). Glottic injury elicited in 27 (67%) of patients, and 11 of them were classified moderate in severity as they had arytenoid granulation tissue and two patients had unilateral ulcerations. Severe glottic injury was described in 6 patients, 4 of them were due to vocal cord immobility and 2 had inter-arytenoid granulations. Subglottic injury was found in 24 patients (60%), and 8 patients were classified as severe due to subglottic granulation tissue Table 3.

**Table 3:** Endoscopic findings in relation to post-extubation stridor

		No PES	PES	*P*-value
**Supraglottic edema**		**13 (65%)**	**17 (40%)**	**0.004**
**Glottic lesions**	**No lesions**	**9 (47.5%)**	**0**	**0.000**
	**Mild lesions**	**7 (37%)**	**1 (5%)**	
	**Moderate**	**2**	**11 (52.5%)**	
	**Severe**	**1**	**5 (30%)**	
**Subglottic**	**No**	**13 (68.5%)**	**3 (14%)**	**0.000**
	**Mild**	**5 (26%)**	**11 (52.5%)**	
	**Moderate**	**0**	**0**	
	**Severe**	**1**	**7 (33.5%)**	
**Vocal cord injury**		**0**	**4 (20%)**	**0.035**
**Tracheo-esopheal fistula**		**0**	**4 (20%)**	**0.035**

### Risk factors for laryngeal injury

Regression analysis of the risk factors of severity of injury showed that the development of ventilator-associated pneumonia (VAP) carried the highest risk (OR 32.111, 95% CI, 5.660 to 182.176), followed by time elapsed till intubation in seconds (OR 11.836, 95% CI 2.889 to 48.490), number of intubation attempts (OR 10.8, CI 2.433 to 47.847), development of pneumothorax (OR 10.231, 95% CI 1.12 to 93.3) (Table 4).

**Table 4:** Logistic regression analysis of the predictors of severity

	***B***	S.E.	Wald	***P*** value	OR (95% C.I. for OR)
Pneumothorax	2.325	1.128	4.250	0.039	10.231 (1.121 to 93.341)
Ventilator-associated pneumonia	3.469	0.886	15.345	0.000	32.111 (5.660 to 182.176)
Days of mechanical ventilation before 1st extubation	0.740	0.229	10.479	0.001	2.096 (1.339 to 3.281)
Intubation attempts	2.378	0.760	9.795	0.002	10.789 (2.433 to 47.847)
Time elapsed	2.471	0.720	11.796	0.001	11.836 (2.889 to 48.490)

However, the tube size, either oral or nasal, and timing of insertion at day or night were not predictive for laryngeal trauma.

## Discussion

The result of this study revealed a high rate of laryngeal lesions in extubated critical pediatric patients. Eighty-two percent of our patients had abnormal findings on laryngobronchoscopy examination. However, the anatomy of the pediatric airway is unique, the pediatric larynx is smaller than the adult larynx, and the subglottic area is the narrowest part [14]. This unique anatomy makes the larynx of children and particularly infants more prone to trauma. Laryngeal injury is multifactorial and analysis of the possible risk factors would help to prevent such complications. We recorded an incidence of PES of 40%. However, laryngeal edema was reported as the most serious and immediate complication of extubation in young children; the incidence of PES in the pediatric critical care is recorded as 2–25% [15]. A study from Brazil reported up to 42% incidence in their PICU, and long duration of ventilation was the main risk factor.

Our study showed that stridor 40% had an association with laryngoscopy findings of moderate to severe glottic and suglottic lesions. The degree of severity of laryngeal injuries could be predicted by the increasing number of intubation attempts, time spent by the physician, development of ventilator complications, and duration of ventilation [16]. Mencke et al. reported less laryngeal morbidity was recorded when the quality of tracheal intubation during anesthesia was good. As for quality improvement, evaluation of the first pass success rate in the emergency department is recommended to monitor the airway performance and subsequently reduce the possibility of laryngeal trauma [17]. VAP was significantly associated with the increased length of PICU stay, mechanical ventilator days, the time of contact of the endotracheal tube to the larynx, and subsequently the laryngeal injury severity [18]. That demonstrates the importance of considering the VAP bundle during the care of mechanically ventilated patients. Data suggest worse outcomes for critically ill patients during nights and weekends compared to weekday staffing models and the influence of the staffing level [12, 19–22]. The tube size was not a risk factor as the tubes used were either 4 or 4.5 mmHg. However, previous studies reported undersedation and duration of ventilation as main risk factors for the development of PES [6, 23].

### Endoscopic findings

Our study detected 50% incidence of granulations and ulcerations of the glottis and subglottic area. Bharti et al. [24] found 97% acute laryngeal injury in his population, of which 88% were significant. A meta-analysis included 29 studies by Brodsky et al. [6] recorded that erythema, edema, ulcerations, and granulations had a frequency of 82%, 70%, 31%, and 27%, respectively. Vocal fold immobility was the most common severe injuries, with a 21% prevalence. The use of cuffed tubes in children below 8 years is still questionable. It was mentioned that the cough leak test has a low sensitivity in the prediction of stridor in two studies [23, 25]. A metanalysis evaluated the use of cuffed versus uncuffed tubes revealed no difference in stridor risk between the two groups, and this study was about a different population of intubated children for anesthesia not critically sick children [5, 26]. However, the ability to predict whom is going to develop stridor and give medications prior to extubation is vital. In addition to the mentioned risk factors, the use of ultrasound measured laryngeal air column width can be helpful [27].

Colton et al. in their cohort study found that the duration of intubation, type, and size of the endotracheal tube had no significant correlation to the incidence of vocal fold mobility and degree of laryngeal injury after prolonged intubation [15].

The current study found the duration of ventilation before the 1st extubation, intubation attempts, and time elapsed during intubation to be independent risk factors for moderate/severe laryngeal injuries.

Similarly, Lilienstein et al. [28] in a large retrospective study showed a longer LOS in the stridor group of patients compared to the matched group. In the study of Barthi et al. [24], only one patient had multiple attempts at intubation. This child developed left vocal fold palsy.

## Conclusion

The incidence of intubation-related laryngeal trauma in pediatric ICU is high and varies widely from mild, non-symptomatic to moderate, and severe with a risk of reintubation and could be predicted when the pediatric patients had already prolonged course of ventilation with the occurrence of complications related to the ventilator and the multiple tube changes or difficult intubation.

## Data Availability

The datasets used and/or analyzed during the current study are available from the corresponding author on reasonable request.

## Abbreviations

CALI: Classification of acute laryngeal injuries
ICU: Intensive care unit
MOSF: Multiple organ system failure
MV: Mechanical ventilation
PES: Post-extubation stridor
TI: Tracheal intubation
VAP: Ventilator-associated pneumonia
